# ﻿Food preference strategy of four sympatric rodents in a temperate forest in northeast China

**DOI:** 10.3897/zookeys.1158.96886

**Published:** 2023-04-21

**Authors:** Dianwei Li, Chengzhi Zhang, Yuwei Cao, Ming Gao, Shiqi Chang, Menghao Xu, Zhimin Jin, Hongwei Ni

**Affiliations:** 1 Heilongjiang Academy of Forestry, No. 134 Haping Road, Harbin, Heilongjiang 150081, China Heilongjiang Academy of Forestry Harbin China; 2 College of Life Sciences and Technology, Mudanjiang Normal University, No. 191 Wenhua Road, Mudanjiang, Heilongjiang 157011, China Mudanjiang Normal University Mudanjiang China; 3 College of Wildlife and Protected Area, Northeast Forestry University, No. 26 Hexing Road, Harbin 150040, China Northeast Forestry University Harbin China

**Keywords:** Coexistence, competition, fitness, food selection, niche, niche differentiation

## Abstract

Rodents are well known as both seed predators and dispersers of various plant species in forest ecosystems, and they play an important role in the regeneration of vegetation. Thus, the research on seed selection and vegetation regeneration by sympatric rodents is an interesting topic. To understand the characteristics of preferences of rodents for different seeds, a semi-natural enclosure experiment was performed with four rodent species (*Apodemuspeninsulae*, *Apodemusagrarius*, *Tscherskiatriton*, and *Clethrionomysrufocanus*) and the seeds of seven plant species (*Pinuskoraiensis*, *Corylusmandshurica*, *Quercusmongolica*, *Juglansmandshurica*, *Armeniacasibirica*, *Prunussalicina*, and *Cerasustomentosa*) to investigate the differentiation in niches and patterns of resource utilization of sympatric rodents. The results showed that all the rodents had consumed many seeds of *Pi.koraiensis*, *Co.mandshurica*, and *Q.mongolica* but differed significantly in how they selected the different seeds. The rate of utilization (*R_i_*) of *Pi.koraiensis*, *Co.mandshurica*, and *Q.mongolica* exhibited the highest values. The *E_i_* values indicated that the rodents tested exhibited differences in their priorities used to select the seeds from different plant species. All four species of rodents exhibited obvious preferences for certain seeds. Korean field mice preferentially consumed the seeds of *Q.mongolica*, *Co.mandshurica*, and *Pi.koraiensis*. Striped field mice favor the seeds of *Co.mandshurica*, *Q.mongolica*, *P.koraiensis*, and Nanking cherry. Greater long-tailed hamsters prefer to consume the seeds of *Pi.koraiensis*, *Co.mandshurica*, *Q.mongolica*, *Pr.salicina*, and *Ce.tomentosa*. *Clethrionomysrufocanus* likes to eat the seeds of *Pi.koraiensis*, *Q.mongolica*, *Co.mandshurica*, and *Ce.tomentosa*. The results supported our hypothesis that sympatric rodents overlap in food selection. However, each rodent species has a marked preference for food selection, and different rodent species differ in their food preferences. This reflects the role of distinct food niche differentiation in their coexistence.

## ﻿Introduction

The coexistence of species and the maintenance of biodiversity are important topics of ecological study. Food is an essential resource for survival, so there are obvious effects of differences in food selection and feeding behavior among animal species on their coexistence. Furthermore, the food choices of animals directly affect their survival and reproduction. However, the factors that affect the selection of food by animals in forest ecosystems are very complicated, and the choices of different rodent species of the seeds from different plant species depend on many factors. The characteristics of the seeds of different plant species vary by size (Vander Wall 2001; Xiao et al. 2005; Chang and Zhang 2014; Luna et al. 2016); the contents of nutrients, tannins, and other secondary metabolites (Steele et al. 1993; Wang and Chen 2012; Chang and Zhang 2014); and the hardness and thickness of the seed coat (Li et al. 2018). All these factors affect the food choices of rodent species and further influence their behavior (Li et al. 2018).

The selective foraging of all animals is a key factor that affects their survival and reproduction. Animals in nature generally exhibit food preference or selective foraging (Hughes and Croy 1993), and no animal uses all the food types that occur in the environment equally, particularly when there are other competing species. In general, animals only prefer a small fraction of the available foods. They rarely feed on most foods and even completely reject some. Effective food preference ensures that animals efficiently intake energy and nutrients and maximize their food selection fitness (Moss 1991; Rogers and Blundell 1991).

Large seeds produced by many plant species are a valuable food source for rodents, which often prefer certain seeds from various plant species within their habitats and are capable of accurately distinguishing the seeds with different characteristics (Vander Wall 2001; Chang and Zhang 2014; Luna et al. 2016; Li et al. 2018). Their identification and choice of food affects the fate of seeds, while the rodents must weigh the input costs (time and energy) when foraging to utilize different eating and dispersal strategies to ensure supplies of optimal food and energy (Vander Wall 1990; Lima 1998; Li et al. 2021).

Seed characteristics, including size and weight (Vander Wall 2001; Luna et al. 2016); seed coat characteristics, such as thickness and hardness (Vander Wall 1990; Luna et al. 2016; Li et al. 2018); seed quality, such as seeds damaged by pests, mildewed, or empty-shelled (Vander Wall 1990; Chang et al. 2010; Vander Wall 2010; Wang et al. 2012); moisture content; nutrients, such as starch, fat, and protein (Vander Wall 1990; Vander Wall 2001); and secondary metabolic compounds, such as tannins and other polyphenolics (Steele et al. 1993; Wang and Chen 2012; Chang and Zhang 2014) affect the decision making of rodents during food selection. Most studies have shown that seed size and nutrients are key factors that determine the foraging strategies of animals, and rodents usually prefer large seeds that are rich in nutrients (Smith and Reichman 1984; Xiao et al. 2006). Harper et al. (1970) strongly suggested that large seeds are favored because they provide a greater return to the seed predators. The optimal foraging theory (OFT; Charnov 1976) suggests that animals favor seeds that generate the largest net return. In general, the total return in foraging increases as the seed size increases, and during the same duration of foraging, the probability that large seeds with higher nutritional content and benefit will be eaten is higher because such foods can better compensate the energy expenditure by the animal during foraging and thus, are more attractive (Xiao et al. 2005; Luna et al. 2016).

The time that seeds are handled is another important factor that influences animal behavior and decision making, and it significantly affects the foraging strategy of rodents (Jacobs 1992; Chang et al. 2010; Li et al. 2020). Because the time of seed handling is associated with the weighing of predation risk against foraging efficiency (Chang et al. 2010; Wang et al. 2012; Li et al. 2020), it tends to be minimized by rodents to enhance survival. The choice of food by rodents is also affected by the size and ability of the individual. A rodent will set an upper threshold for the preferred seed size based on its own size, so seeds from the same plant species may provide very different net returns to varying predators (Jacobs 1992; Luna et al. 2016). Because of differences in body size, strength and mouth type, predators differ in their ability to handle different seeds.

Studies of animal food choice are significant to understand the co-evolution of animal and plant systems, as well as niche differentiation among sympatric animals (Vander Wall 2001; Li and Zhang 2003; Vander Wall and Beck 2011; Li et al. 2018). Since rodents are the primary predators and dispersers of seeds in forest ecosystems (Vander Wall 1990), they form a system of foraging and reciprocity with seeds. The degree of preference of rodents for a certain type of seed and the differential selection of different seeds can lead to changes in predation pressure and the rate of dispersion among plant species (Pons and Pausas 2006), and it may have important impacts on seed dispersal and the natural regeneration of forest communities through the heavy consumption of seeds by animals (Vander Wall 1990; Perea et al. 2011; Li et al. 2021). Their consumption can distribute seeds and lead to seedling establishment as a result of hoarding behavior (Janzen 1971; Grubb 1977; Clark and Clark 1984; Willson and Whelan 1990; Vander Wall 2001; Briggs et al. 2009). The seed mortality owing to the foraging of animals affects the fitness, population structure, and composition of species of the plant community (Willson and Whelan 1990). The competition for resources among sympatric animals has been an important topic in community ecology, and numerous studies have shown that competition is the primary factor that causes variation in the use of resources by animals, thus, leading to differences in morphology and behavioral strategies among species.

Currently, the differential selection of seeds of a given plant species by sympatric rodent species has not been well researched. In this study, we used four sympatric rodent species and the seeds of seven plant species distributed in northern temperate forests to investigate the differences in seed choice related to the species under semi-natural enclosure conditions, as well as the effect of seed characteristics on the strategic differentiation in food choice by rodents to gain insight into the niche partitioning of sympatric rodents and its effect on the fate of the seeds of specific plants and vegetation renewal. We hypothesized that sympatric rodents feed on largely similar food items but differ significantly in their preferred choices.

## ﻿Materials and methods

### ﻿Study site and species

This study was conducted from April to June in 2019 in the Zhang Guangcai Mountains in Mudanjiang (elevation 400 to 900 m; 44°47'N, 129°07'E) located in Heilongjiang Province, northeast China. The research area is located north of the Changbai Mountain. The climate at the site is dominated by the northern temperate zonal continental monsoon climate, with four distinct seasons and a short frost-free period of approximately 90–115 days. The annual average air temperature is 4.3 °C, with a maximum of 34.4 °C and a minimum of -39 °C. The average annual precipitation is approximately 670 mm. The representative vegetation in the study area primarily includes secondary broad-leaf forest and coniferous broad-leaf forest. At the study site, four rodent species, *Apodemuspeninsulae* (Thomas, 1907), *Apodemusagrarius* (Pallas, 1771), *Tscherskiatriton* (De Winton, 1899), and *Clethrionomysrufocanus* (Sundevall, 1846), rely on seeds as important food sources. Seven sympatric seeds, including *Pinuskoraiensis* (Siebold & Zuccarini, 1861), *Corylusmandshurica* (Maximowic, 1856), *Quercusmongolica* (Fischer & Ledebour, 1850), *Juglansmandshurica* (Maximowic, 1856), *Armeniacasibirica* (Lamarck, 1783), *Prunussalicina* (Lindley, 1830), and *Cerasustomentosa* (Thunberg) Masamune and S. Suzuki 1936), were used in the experiment. Fresh seeds were collected during the fruiting season and then dried naturally at field temperatures until use.

### ﻿Capture of live animals by cage trapping

Live rodent samples were caught through cage trapping. In each trap (30 cm × 25 cm × 20 cm), fried pumpkin (*Cucurbita* spp.) seeds (for food) and carrots (*Daucuscarota* var. *sativa* Hoffmann, 1791) (for water) were placed as bait, and cotton rolls were provided as denning material. The cages were placed along two transects in the sample plot at 20 m spaces with one cage per 20 m × 5 m. On the second day after the cages were placed, the captures were examined, and pregnant females and juveniles were immediately released. Captured adult rodents were transferred to terraria (65 cm × 35 cm × 25 cm) covered with wire mesh and supplied with drinking water and a suitable amount of litter, and the terraria were placed in the natural environment.

### ﻿Seed preference sequence experiment

The captured rodents were allowed one day of acclimation to the new environment and the seeds. Plump seeds were randomly selected from each of seven plant species, and one seed from each species was placed on the feeding plate in a terrarium occupied by one rodent. The order in which the rodent took the seeds was monitored using a video camera. Each rodent was tested for more than three hours, during which all the seeds were consumed, or the rodent no longer selected the seeds. The seeds for experiments were replaced, and the experimental process was repeated. After the experiment had been repeated three times for each rodent, the terrarium was cleaned, and the experimental animal was replaced with another individual. Four sympatric rodent species, i.e., *A.peninsulae* (*n* = 16), *A.agrarius* (*n* = 10), *T.triton* (*n* = 10), and *Cl.rufocanus* (*n* = 9), and a total of 135 seeds from seven plant species was used in the experiment.

### ﻿Seed preference experiment

Four semi-natural enclosures (1 m × 1 m × 1 m) were constructed on relatively flat terrain in the study area. At one corner of each enclosure, a den was made that contained some cotton rolls to keep the rodent warm, and a small water container was set up and replenished regularly to allow the rodent to drink freely. A feeding plate was placed in the center of the enclosure, which was where the seeds were supplied for the experimental rodent.

Based on the body size of the rodents and their food consumption observed in the previous experiment, one *J.mandshurica* seed and two seeds of the remaining plant species were supplied in tests that involved *A.peninsulae*, *A.agrarius*, and *Cl.rufocanus*, and those that involved *T.triton*. Three seeds from each plant species were supplied to eliminate the effect of consumption capacity and body size of the species of rodent. The rationale was to supply an appropriate amount of food to avoid the situation in which the rodent took all the seeds owing to insufficient food supply or the situation in which it only consumed its most favorite seeds from one or two plant species but not the seeds from other plants. The data from the individuals that died during the experiment were excluded, so 26 *A.peninsulae*, 17 *A.agrarius*, 10 *T.triton*, and 14 *Cl.rufocanus* were tested using 432 seeds from each of six plant species, i.e., *Pi.koraiensis*, *Co.mandshurica*, *Q.mongolica*, *A.sibirica*, *Pr.salicina*, *Ce.tomentosa*, and 261 seeds from *J.mandshurica*.

### ﻿Electivity index

The Ivlev electivity index (*E_i_*) (Scarlett and Smith 1991) was used to describe the food preference. Its formula is as follows: *E_i_* = (*R_i_* – *P_i_*) / (*R_i_* + *P_i_*) in which the seed utilization rate *R_i_* = (the total number of seeds from the *i*^th^ species that have been consumed/ the total number of seeds from all species that have been consumed) × 100%; and the rate of seed availability *P_i_* = (the total number of seeds from the *i*^th^ species that have been supplied/ the total number of seeds from all species supplied) × 100%. The *E_i_* ranges from -1 to 1> if *E_i_* > 0, the rodent positively selects for the seeds from the species. If *E_i_* < 0, the rodent negatively selects. If *E_i_* = 0, the rodent exhibits no preference for the seeds, and if *E_i_* = -1, the rodent makes no choice at all. Based on the *E_i_* value, the preference of rodents for the seeds was categorized into four levels: strongly preferred (*E_i_* ≥ 0.5), preferred (*E_i_* > 0), barely ate (*E_i_* > -0.5), and avoided (*E_i_* ≥ -1).

### ﻿Statistical analysis

Data processing and statistical analyses were performed with Microsoft Excel 2007 (Redmond, WA, USA) and SPSS 21.0 (IBM, Inc., Armonk, NY, USA). The data were subjected to the Kolmogorov-Smirnov normality and Homogeneity-of-variance tests before processing, and in the case that the data did not comply with parametric assumptions, nonparametric tests were conducted. The Kruskal-Wallis H test was used to compare the differences in the choice of seeds from different plant species by the same species of rodent, and the Mann-Whitney U test was used to perform pair-wise comparisons of the differences in food choice between different species of rodents. Descriptive statistics were expressed as the mean ± SD. The level of statistical significance was set to α = 0.05, and high statistical significance was set to α = 0.01.

## ﻿Results

### ﻿Feeding and utilization of the seeds

The consumption of food differed significantly among the four species of rodents. The amount of all the seeds taken each time by *T.triton* was 16.57 ± 2.47 (*n* = 30), which was significantly higher than those of *A.peninsulae* (5.65 ± 2.43, *n* = 78), *Cl.rufocanus* (4.88 ± 2.05, *n* = 42), and *A.agrarius* (4.65 ± 2.44, *n* = 51). *Apodemuspeninsulae* consumed significantly more seeds than *A.agrarius*. The species also showed differences in the amounts of each type of seed consumed at each feeding.

The four species of rodent exhibited varying total rates of consumption (*r_i_*) on different seeds, but they all had a higher total rate of consumption on the seeds of *Pi.koraiensis*, *Co.mandshurica*, and *Q.mongolica*. Among them, *A.peninsulae* consumed 85.90% of the *Q.mongolica* seeds, 67.95% of the *Co.mandshurica* seeds, and 64.10% of the *Pi.koraiensis* seeds. *Apodemusagrarius* consumed 61.76% of the *Co.mandshurica* seeds, 57.84% of the *Q.mongolica* seeds, and 54.90% of the *Pi.koraiensis* seeds. *Tscherskiatriton* consumed 100% of the seeds of *Pi.koraiensis*, *Co.mandshurica*, and *Q.mongolica*, and in addition to the *J.mandshurica* seeds, *T.triton* consumed more of the seeds of other plant species than the other species of rodents (*Pr.salicina*: 88.89%; *Ce.tomentosa*: 84.44%; *A.sibirica*: 77.78%). *Cl.rufocanus* consumed 65.31% of the *Pi.koraiensis* seeds, 54.08% of the *Q.mongolica* seeds, and 47.96% of the *Co.mandshurica* seeds (Table 1).

**Table 1.** Statistical data and analysis of the feeding and utilization of seven species of seeds by four rodents.

Rodent species	Index	*Pinuskoraiensis*	*Corylusmandshurica*	*Quercusmongolica*	*Juglansmandshurica*	*Armeniacasibirica*	*Prunussalicina*	*Cerasustomentosa*	Kruskal-Wallis H test
*Apodemuspeninsulae* (*n* = 26)	*SN*	2	2	2	1	2	2	2	
	*CN*	1.28±0.91	1.36±0.82	1.72±0.64	0.23±0.42	0.78±0.82	0.28±0.58	0±0	χ*^2^*=247.897, df=6, *P*<0.001
	*TS*	156	156	156	78	156	156	156	
	*TC*	100	106	134	18	61	22	0	
	*r_i_* (%)	64.10	67.95	85.90	23.08	39.10	14.10	0	χ*^2^*=219.514, df=6, *P*<0.001
	*R_i_* (%)	22.68	24.04	30.39	4.08	13.83	4.99	0	χ*^2^*=247.897, df=6, *P*<0.001
	*E_i_*	0.192	0.219	0.328	-0.307	-0.053	-0.510	-1.000	χ*^2^*=219.514, df=6, *P*<0.001
*Apodemusagrarius* (*n* = 17)	*SN*	2	2	2	1	2	2	2	
	*EN*	1.10±0.92	1.24±0.86	1.16±0.70	0±0	0.08±0.34	0.29±0.61	0.78±0.88	χ*^2^*=129.378, df=6, *P*<0.001
	*TS*	102	102	102	51	102	102	102	
	*TC*	56	63	59	0	4	15	40	
	*r_i_* (%)	54.90	61.76	57.84	0	3.92	14.71	39.21	χ*^2^*=129.378, df=6, *P*<0.001
	*R_i_* (%)	23.63	26.58	24.89	0	1.69	6.33	16.88	χ*^2^*=129.636, df=6, *P*<0.001
	*E_i_*	0.211	0.267	0.236	-1.000	-0.802	-0.417	0.046	χ*^2^*=126.897, df=6, *P*<0.001
*Tscherskiatriton* (n = 10)	*SN*	3	3	3	3	3	3	3	
	*CN*	3.00±0.00	3.00±0.0	3.00±0.0	0.03±0.18	2.33±1.09	2.67±0.88	2.53±1.04	χ^2^=136.548, df=6, *P*<0.001
	*TS*	90	90	90	90	90	90	90	
	*TC*	90	90	90	1	70	80	76	
	*r_i_* (%)	100	100	100	1.11	77.78	88.89	84.44	χ*^2^*=136.548, df=6, *P*<0.001
	*R_i_* (%)	18.11	18.11	18.11	0.2	14.08	16.10	15.29	χ*^2^*=136.548, df=6, *P*<0.001
	*E_i_*	0.118	0.118	0.118	-0.972	-0.007	0.060	0.034	χ*^2^*=136.548, df=6, *P*<0.001
*Clethrionomysrufocanus* (*n* = 14)	*SN*	2	2	2	1	2	2	2	
	*CN*	1.48±0.55	1.07±0.89	1.19±0.74	0.00±0.00	0.07±0.26	0.31±0.60	0.76±0.88	χ*^2^*=132.491, df=6, *P*<0.001
	*TS*	84	84	84	42	84	84	84	
	*TC*	62	45	50	0	4	15	34	
	*r_i_* (%)	73.81	53.57	59.52	0	3.57	15.48	38.10	χ*^2^*=132.491, df=6, *P*<0.001
	*R_i_* (%)	30.24	21.95	24.39	0	1.46	6.34	15.61	χ*^2^*=132.491, df=6, *P*<0.001
	*E_i_*	0.326	0.176	0.226	-1.000	-0.826	-0.416	0.007	χ*^2^*=132.491, df=6, *P*<0.001
Kruskal-Wallis H test	*R_i_*, df=3	χ^2^=85.670 *P*<0.001	χ^2^=40.321 *P*<0.001	χ^2^=57.718 *P*<0.001	χ^2^=28.199 *P*<0.001	χ^2^=84.937 *P*<0.001	χ^2^=74.190 *P*<0.001	χ^2^=74.744 *P*<0.001	
	*E_i_*, df=3	χ^2^=24.924 *P*<0.001	χ^2^=15.581 *P*=0.001	χ^2^=34.367 *P*<0.001	χ^2^=28.199 *P*<0.001	χ^2^=65.362 *P*<0.001	χ^2^=43.913 *P*<0.001	χ^2^=67.271 *P*<0.001	

* *SN* (Supply number): the number of s eeds supplied each time. *CN* (Consumption number): the average number of seeds consumed. *TS* (Total supply number): the total number of seeds from the *i*^th^ species that had been supplied, *TS* = ∑*SN*. *TC* (Total consumption number): the total number of seeds from the *i*^th^ species that had been selected. *TC* = ∑*CN*. *r_i_* (the rate of consumption of seeds from the *i*^th^ species), *r_i_* = (*TC_i_* / *TS_i_*) × 100%. *R_i_* (the rate of utilization of seeds from the *i*^th^ species), *R_i_* = (*TC_i_* / ∑*TC*) × 100%. *P_i_* (the rate of availability of seeds from the *i*^th^ species), *P_i_* = (*TS_i_* / ∑*TS*) ×100%. *E_i_* (the Ivlev electivity index), *E_i_* = (*R_i_* – *P_i_*) / (*R_i_* + *P_i_*).

**Figure 1.**
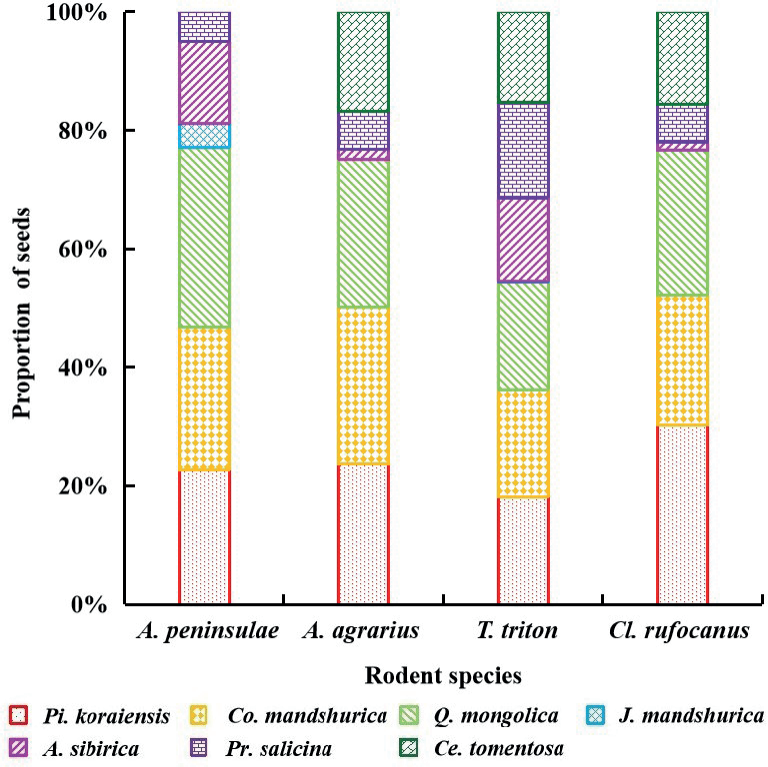
The food spectrum of four rodents. In each bar chart, different types of shading represent the proportion of different seeds.

In terms of *R_i_*, *Pi.koraiensis*, *Co.mandshurica*, and *Q.mongolica* exhibited the highest values (*A.peninsulae*: 77.11%; *A.agrarius*: 75.10%; *T.triton*: 54.33%; *Cl.rufocanus*: 76.58%). The seeds of *A.sibirica*, *Pr.salicina*, and *Ce.tomentosa* accounted for 45.47% of the food spectrum of *T.triton* (Fig. 1).

### ﻿Priority of seed selection

**Figure 2.**
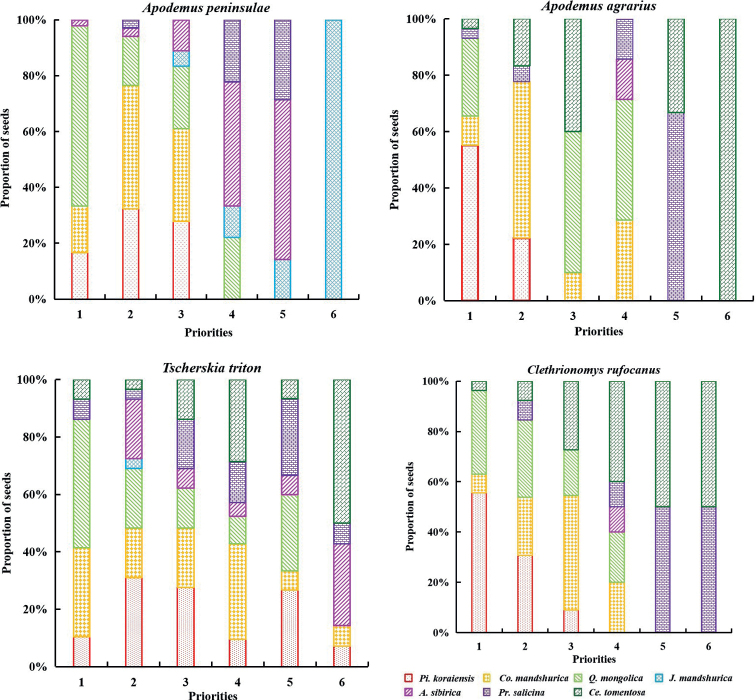
The priorities of seven seeds were selected by four rodents *Apodemuspeninsulae*, *A.agrarius*, *Tscherskiatriton*, *Clethrionomysrufocanus*. 1–6: The order of seeds that were selected. In each bar chart of 1–6, different types of shading represent the proportion of different seeds as in the color key.

The rodents tested exhibited differences in the priority by which they selected the seeds from different plant species. For *A.peninsulae*, the order was *Q.mongolica* > *Co.mandshurica* > *Pi.koraiensis* > *A.sibirica* > *Pr.salicina* > *J.mandshurica* > *Ce.tomentosa*. For *A.agrarius*, the order was *Pi.koraiensis* > *Q.mongolica* > *Co.mandshurica* > *Ce.tomentosa* > *Pr.salicina* > *A.sibirica* > *J.mandshurica*. For *T.triton*, the order was *Q.mongolica* > *Co.mandshurica* > *Pi.koraiensis* > *Pr.salicina* > *Ce.tomentosa* > *A.sibirica* > *J.mandshurica*. Finally, for *Cl.rufocanus*, the order was *Pi.koraiensis* > *Q.mongolica* > *Co.mandshurica* > *Ce.tomentosa* > *Pr.salicina* > *A.sibirica* > *J.mandshurica* (Fig. 2).

### ﻿Rodent choices of different seeds

The *E_i_* values indicate that the four rodent species differed in their choices of seeds.

*Apodemuspeninsulae* obviously preferred certain seeds (*E_i_* : χ*^2^* = 219.514, df = 6, *P* < 0.001) of *Q.mongolica*, *Co.mandshurica*, and *Pi.koraiensis* (*E_i_* : 0.328, 0.219, 0.192). It rarely ate the seeds of *A.sibirica* or *J.mandshurica* (*E_i_*: -0.053, -0.307) and avoided those of *Pr.salicina* and *Ce.tomentosa* (*E_i_* : -0.510, -1.000). The favored seeds were consumed at significantly higher levels than the others (*P* < 0.001). The species consumed more seeds of *Q.mongolica* than those of *Pi.koraiensis* or *Co.mandshurica* (*U* = 2300.000, *P* = 0.001; *U* = 2314.000, *P* = 0.001), and it favored the seeds of *A.sibirica* over those of *J.mandshurica* or *Pr.salicina* (*U* = 2010.500, *P* < 0.001; *U* = 2313.000, *P* = 0.003). It did not show a preference between the seeds of *J.mandshurica* and *Pr.salicina* (*U* = 2895.000, *P* = 0.474).

*Apodemusagrarius* noticeably preferred certain seeds (*E_i_* : *χ^2^* = 126.897, df = 6, *P* < 0.001), those of *Co.mandshurica*, *Q.mongolica*, *Pi.koraiensis*, and *Ce.tomentosa* (*E_i_*: 0.267, 0.236, 0.211, 0.046). It rarely ate the seeds of *Pr.salicina* (*E_i_* : -0.417), and it avoided those of *A.sibirica* and *J.mandshurica* with a significantly higher consumption of the favored seeds over the others (*P* < 0.05). The species consumed more seeds of *Co.mandshurica* or *Q.mongolica* than those of *Ce.tomentosa* (*U* = 928.500, *P* = 0.008 < 0.05; *U* = 948.500, *P* = 0.013 < 0.05), and it favored the seeds of *Pr.salicina* over those of *A.sibirica* (*U* = 1105.00, *P* = 0.029).

*Tscherskiatriton* exhibited obvious preferences for certain seeds (*E_i_* : *χ^2^* = 136.548, df = 6, *P* < 0.001), consuming the seeds of *Pi.koraiensis*, *Co.mandshurica*, *Q.mongolica*, *P.salicina*, and *Ce.tomentosa* (*E_i_*: 0.118, 0.118, 0.118, 0.060, 0.034) and rarely ate those of *A.sibirica* (*E_i_*:-0.007). This rodent entirely avoided those of *J.mandshurica* (*E_i_*: -0.972). The species consumed more seeds of *Pi.koraiensis*, *Co.mandshurica*, and *Q.mongolica* than those of *Pr.salicina* (*U* = 390.000, *P* = 0.040) or *Ce.tomentosa* (*U* = 360.000, *P* = 0.011), but it showed no preference among the seeds of *Pi.koraiensis*, *Co.mandshurica*, and *Q.mongolica* or among those of *P.salicina*, *Ce.tomentosa*, and *A.sibirica* (*U* = 366.000, *P* = 0.93; *U* = 420.000, *P* = 0.494; *U* = 396.000, *P* = 0.304). However, the seeds of *J.mandshurica* (*P* < 0.001) were the least preferred.

*Clethrionomysrufocanus* exhibited obvious preferences for certain seeds (*E_i_*: *χ^2^* = 132.491, df = 6, *P* < 0.001) and preferentially consumed the seeds of *Pi.koraiensis*, *Q.mongolica*, *Co.mandshurica*, and *Ce.tomentosa* (*E_i_*: 0.326, 0.226, 0.176, 0.007), while it rarely ate those of *Pr.salicina* (*E_i_*: -0.416). It avoided those of *A.sibirica* and *J.mandshurica* (*E_i_*: -0.826, -1.000). Among its favored seeds, the species consumed more seeds of *Pi.koraiensis* than of *Co.mandshurica* (*U* = 673.500, *P* = 0.043 < 0.05) and more seeds of *Pi.koraiensis* and *Q.mongolica* than those of *Ce.tomentosa* (*U* = 477.000, *P* < 0.001; *U* = 632.000, *P* = 0.018 < 0.05). It exhibited no significant difference in selecting the seeds of *Q.mongolica*, *Pi.koraiensis*, and *Co.mandshurica* (*U* = 706.000, *P* = 0.082, *U* = 825.000, *P* = 0.587) while it showed no preference between the seeds of *Co.mandshurica* and *Ce.tomentosa* (*U* = 717.000, *P* = 0.112).

### ﻿Rodent choices of seeds from the same plant species

The four rodent species exhibited differences in the choice of seeds from the same plant species (Kruskal-Wallis H, *E_i_*: df = 3, *P* < 0.05; Table 1).

*Clethrionomysrufocanus* preferred the seeds of *Pi.koraiensis* the most of those studied and had similar preferences to *A.agrarius* (*U* = 956.500, *P* = 0.365). They were higher than those of *A.peninsulae* (*U* = 986.000, *P* < 0.001) or *T.triton* (*U* = 30.000, *P* < 0.001).

For the seeds of *Co.mandshurica*, the preference by *A.agrarius* was the greatest, similar to that of *Cl.rufocanus* (*U* = 1447.500, *P* = 0.275) and higher than that of *A.peninsulae* (*U* = 1468.000, *P* = 0.009) or *T.triton* (*U* = 420.000, *P* < 0.001).

For the seeds of *Q.mongolica*, *A.peninsulae* liked them the most and had levels of preference similar to that of *A.agrarius* (*U* = 1112.000, *P* = 0.152) and *Cl.rufocanus* (*U* = 1532.000, *P* = 0.525), while that of *T.triton* was significantly lower than that of each of the remaining three species (*A.peninsulae*: *U* = 420.000, *P* < 0.001; *A.agrarius*: *U* = 480.000, *P* < 0.001; *Cl.rufocanus*: *U* = 240.000, *P* < 0.001) .

The preference of *A.peninsulae* for *J.mandshurica* seeds was significantly greater than that of each of the other rodent species (*A.agrarius*: *U* = 1530.000, *P* < 0.001; *A.agrarius*: *U* = 930.000, *P* = 0.013; *Cl.rufocanus*: = 1260.000, *P* = 0.001).

For the seeds of *A.sibirica*, *T.triton* most strongly preferred these seeds, and it significantly preferred them than each of the other rodent species (*A.peninsulae*: *U* = 872.000, *P* = 0.034 < 0.05; *A.agrarius*: *U* = 186.000, *P* < 0.001; *Cl.rufocanus*: *U* = 168.000, *P* < 0.001); that of *A.peninsulae* was also higher than that of *A.agrarius* (*U* = 1060.000, *P* < 0.001) or *Cl.rufocanus* (*U* = 879.000, *P* < 0.001).

For the seeds of *Pr.Salicina*, the preference by *T.triton* was the greatest and significantly higher than that of each of the other rodent species (*A.peninsulae*: *U* = 259.000, *P* < 0.001; *A.agrarius*: *U* = 370.000, *P* < 0.001; *Cl.rufocanus*: *U* = 332.000, *P* < 0.001), among which there was no difference in preference (Mann-Whitney U, *E_i_*: *P* > 0.05).

For the seeds of *Ce.tomentosa*, three rodent species, *A.agrarius*, *T.triton*, and *Cl.rufocanus* exhibited no differences in preferences; *A.peninsulae* exhibited no choice at all (Mann-Whitney U, *E_i_*: *P* > 0.05).

## ﻿Discussion

Analyses of food consumption, the order of seed selection, and *E_i_* demonstrated that the four rodent species in this study all favored or preferred the seeds of *Pi.koraiensis*, *Co.mandshurica*, and *Q.mongolica*. These seeds are large and commonly found in boreal forests that have high numbers of *J.mandshurica* of the appropriate sizes, easily handled, and containing abundant resources. Thus, they have become a favored food for most species of small rodents during the long natural process of evolution. Furthermore, the long history of competition has led to food niche differentiation in sympatric rodents, whose preference for the seeds of certain plant species has largely been demonstrated in this work. Among the three plant species most favored, *A.peninsulae* preferred the seeds of *Q.mongolica*; *A.agrarius* preferred the seeds of *Co.mandshurica*, and *Cl.rufocanus* preferred the seeds of *Pi.koraiensis*. *Tscherskiatriton* exhibited no preference. The preferences of the four rodent species for the seeds of different plant species reflect a mutually beneficial symbiotic relationship that has evolved over a long time. As food resources, the seeds of these plants provide the necessary nutrients for the survival and reproduction of these rodents and thus, affect animal behavior and population dynamics (Janzen 1971; Vander Wall 2001; Luna et al. 2016; Li et al. 2018). The foraging, transportation, and hoarding of plant seeds and fruits by these rodent species influences the spread and renewal of vegetation (Janzen 1971; Grubb 1977; Clark and Clark 1984; Willson and Whelan 1990; Vander Wall 2001; Briggs et al. 2009; Li et al. 2021).

Intrinsic factors of a rodent, such as its body size and ability to process food, also exert important effects on its food selection. Among the four rodent species tested in this study, *T.triton* is the largest in body size followed by *A.peninsulae*, while *A.agrarius* and *Cl.rufocanus* are smaller. The large-sized *T.triton* therefore has a higher capacity for handling food and consumed all the seeds of *Pi.koraiensis*, *Co.mandshurica*, and *Q.mongolica*, as well as most of the seeds of *A.sibirica*, *Pr.salicina*, and *Ce.tomentosa*. Furthermore, its *E_i_* values demonstrated that it favors seeds from additional plant species, indicating that the diet breadth and eating capacity of *T.triton* are higher than those of each of the other three rodent species. The food preference of *T.triton* was not as refined as that of other species, possibly because the supply of its favored seed resources is too small to meet its large demand for food, so it must exploit other seed resources.

According to the Optimal Foraging Theory, natural selection has enabled animals to maximize their net benefits during foraging, and the most efficient foraging strategy ensures survival and reproductive success (Charnov 1976). Many small rodent species have been found to not feed on the seeds of *J.mandshurica*, and *A.peninsulae* only consumed a small percentage (23.08%) of these seeds. This resulted in an *R_i_* that only accounted for 4.08% of its total food consumption. This result is related to the seed-handling ability of the rodents. Although the seeds of *J.mandshurica* are rich in nutrients and can provide more benefit in a single seed, they are large and have a hard seed coat, which pose substantial challenges to small rodents during both transport and consumption, making it difficult for them to substantially benefit from the seeds (Xiao et al. 2005). This is consistent with the result of our field studies (unpublished results) that showed the small rodents rarely chose the seeds of *J.mandshurica*. The preferences of the four rodent species in this study for the seeds of *A.sibirica*, *Pr.salicina*, and *Ce.tomentosa*, three sparsely distributed plant species, varied markedly, and the consumption of *Ce.tomentosa* seeds by *A.agrarius* and *Cl.rufocanus* was greater than that of *A.sibirica* or *Pr.salicina*, likely owing to differences in the seed-handling abilities of the different species. *Apodemusagrarius* and *Cl.rufocanus* are the smallest rodents and therefore, must invest tremendous effort to handle the seeds of *A.sibirica* and *Pr.salicina* with their thick, hard seed coats, whereas it is easier for them to handle the smaller seeds of *Ce.tomentosa*. However, we found that *A.peninsulae* did not feed on the seeds of *Ce.tomentosa*, which could be because these seeds are too small to provide sufficient food resources.

According to the principle of competitive exclusion, competitors for the same limiting resource cannot coexist, but it is very difficult to directly observe competition in nature, particularly in the cases of interspecific and intraspecific competition in rodents. The food selection results of this study indicate that the four sympatric rodent species compete with each other for food and could have a high degree of niche overlap for the same food resources. However, this study did not account for factors such as differences in the levels of resource availability and competition, which are the outcome of long-term adaptation of the animals to their natural environment, and thus reflect their potential patterns of food resource niche differentiation. Niche differentiation avoids competition and enables sympatric species to coexist despite limited resources, thus enriching biodiversity and being necessary to sustain the coexistence of species (Kartzinel et al. 2015). Such niche differentiation also depends on the different habitats or microhabitats in which the animals live. *Apodemuspeninsulae* is the dominant species in the broad-leaved coniferous and broad-leaved mixed forests in the north. *Apodemusagrarius* is primarily distributed in the purlieus of forests. *Tscherskiatriton* occupies various habitats but dominates in grassland, farmland, and hilly areas, while *Cl.rufocanus* is primarily distributed in coniferous forest habitats. Moreover, feeding niche differentiation does not necessarily indicate the absence of competition, which is related to the amount of food resources. Abundant food resources enable greater interspecies niche overlap, whereas scarce food resources lead to competition (Lawlor 1980).

## ﻿Conclusions

The characteristics of seeds and the intrinsic factors of the rodents exert important effects on the food selection. Rodents can identify different seed properties of the sympatric distribution and form specific feeding preferences. The four rodents all favored the seeds of *Pi.koraiensis*, *Co.mandshurica*, and *Q.mongolica* in a temperate forest in northeast China. Therefore, there are different degrees of overlap in food selection among the sympatric species of rodents because of different degrees of shared food preferences. In order to avoid excessive sympatric competition, rodents adjust their food preference strategies to differentiate feeding niches and thus achieve coexistence.
